# Victim-centered justice through profiling: clustering analysis of parent who suffered abuse

**DOI:** 10.3389/fpsyg.2026.1694603

**Published:** 2026-05-06

**Authors:** C. Moret-Tatay, M. J. Beneyto-Arrojo, I. Iborra-Marmolejo, C. Mosquera-Ordónez, A. Jiménez-Ribera, S. Sempere-Faus

**Affiliations:** Catholic University of Valencia San Vicente Mártir, Valencia, Spain

**Keywords:** cluster analysis, filio-parental violence, justice, parent abuse, victims

## Abstract

Understanding victimization in the context of parent abuse requires a comprehensive approach that considers the complex interplay of demographic, situational, and psychological factors. This study examines 3,834 administrative records from a victim support system, corresponding to 782 unique cases of parents who suffered violence, to identify distinct victim typologies and inform more effective, victim-centered interventions within the legal system. Drawing on variables such as age, gender, marital status, socioeconomic background, and harm types (physical, psychological, economic, and social), the dataset was analyzed using K-means clustering to uncover latent patterns. A two-cluster solution was selected based on statistical validation, offering both interpretability and strong group separation. Cluster 0, comprising 26.3% of the sample, was characterized by a slightly older average age (mean = 61.3 years) and exhibited a diverse set of vulnerability factors not primarily defined by psychological harm resulting from criminal acts. Members of this cluster showed elevated rates of widowhood (28.6% compared to 17.7% in Cluster 1), suggesting potentially different social support needs. In contrast, Cluster 1, labeled Psychological-Impact Victims, constituted the majority of the sample (73.7%), defined by the presence of psychological harm following criminal incidents, with a lower average age (mean = 58.0 years) and higher rates of separation or divorce (26.9% vs. 19.4%). This binary classification reveals a meaningful distinction in victim experiences of parent who suffered violence. Cluster 0 requires broad social support, while Cluster 1 needs trauma-informed psychological care. These findings emphasize the importance of tailored interventions over uniform approaches.

## Introduction

The literature employs a vast variety of terms to describe violence perpetrated by sons or daughters against their parents. The most frequently adopted designations include child-to-parent violence and parent abuse (Ibabe, 2020), both referring to repeated behaviors by a child aimed at inflicting physical, psychological, or economic harm on a parent. However, much of the terminology used in this field focuses on violence perpetrated by minors or adolescents, although definitions vary regarding the specific age cut-off. Terms such as “child-to-parent violence” or “adolescent-to-parent aggression” frequently exclude instances involving adult children who assault their parents (Rogers and Ashworth, 2024).

Although childhood aggression has seen a marked rise in international research in recent years (Cano-Lozano et al., 2023), becoming one of the most extensively studied topics within the field of family violence (Liu et al., 2013; Rutter, 2023), there remains a notable lack of studies focusing on perpetrators over the age of 18, who are legally considered adults (Edenborough et al., 2011; Gámez-Guadix and Calvete, 2012; Erostarbe et al., 2018; Simmons et al., 2019a,b). In defining “parent abuse”, cohabitation between the perpetrator and the parent should be prioritized over the use of an arbitrary age threshold. Importantly, parent abuse does not cease once children reach adulthood, and adult perpetrators may face more severe legal consequences than their younger counterparts. Therefore, further research into abuse perpetrated by adult children against their parents is warranted (Ibabe, 2020).

Unlike other forms of domestic violence, the emotional bond between perpetrators and victims in child-to-parent violence creates dynamics of ambivalence, guilt, shame, and reluctance to report the abuse (Hernández et al., 2020). The progressive and bidirectional nature of many of these situations often makes them difficult to recognize and label as violence. Harm may extend beyond the immediate incident, leading to long-term physical injuries, psychological disorders such as PTSD, economic instability, and social isolation (Thomas and Kopel, 2023). This phenomenon may manifest during both adolescence and adulthood of the children, taking physical, psychological, economic, or symbolic forms that deeply undermine victims' mental health and well being (Erostarbe et al., 2018). Research indicates that victimized parents frequently exhibit high levels of anxiety, depression, chronic stress, and social isolation, which hinders their access to protection and support resources (Dahouri et al., 2025). Furthermore, the criminal justice process itself can exacerbate psychological harm if victims are not adequately supported (Herman, 2005).

Despite the growing academic interest in this type of abuse, significant gaps remain in the specific analysis of victims, who are often rendered invisible in institutional interventions (Cano-Lozano et al., 2023). Justice therefore requires a comprehensive understanding of the multifaceted dimensions of victimization, particularly within the legal system (Mishra, 2019). Victimology, as an evolving discipline, seeks to develop general principles concerning the causes of victimization, its processes and consequences, and the efficacy of prevention and intervention strategies (Ballard and Kubrin, 2023). Addressing victims' needs demands a nuanced approach that accounts for both individual and contextual factors (Manikis, 2019).

Victims' experiences are shaped by demographic variables such as age, gender, marital status, socioeconomic background, and educational attainment, which influence vulnerability to crime as well as access to justice, resources, and support systems (Fohring, 2022). For example, children and the older adult often face unique challenges in navigating legal proceedings and recovering from trauma (Díaz-Faes and Pereda, 2020). Gender plays a central role, as women frequently experience specific forms of violence that require tailored support services. Marital status, number of dependents, and socioeconomic disadvantage may further affect access to assistance and legal recourse.

Understanding the nature of the offense itself is equally essential. Variables such as the type of offense, use of weapons, and the relationship between victim and offender significantly influence the severity of harm and the pathways to justice. The presence of aggravating circumstances and the degree of inflicted physical, psychological, economic, or social harm must be considered when designing support strategies (Roberts, 2009). A responsive justice system must therefore incorporate these diverse variables into its practices. Victim impact statements, shown to enhance trust in the legal system, offer a platform for victims to express their experiences and needs (Laxminarayan, 2015). Legal aid, psychological counseling, and financial compensation further empower victims and facilitate recovery.

Recent proposals have emphasized restorative justice approaches aimed at facilitating dialogue and healing between victims and offenders (Nascimento et al., 2022). However, critiques persist regarding the inclusion of victim input in sentencing, which may result in disproportionate punitive measures (Doak and O'Mahony, 2006). Beyond restorative and retributive frameworks, additional models highlight that victim-centered justice must reflect the complexity of victim experiences and support systems (Daly, 2017; Puspitosari and Priambada, 2018; Silberstein, 2018; Wemmers, 2002). Tailoring interventions to diverse needs enhances both individual recovery and the legitimacy of the justice system.

In this context, identifying differentiated victim profiles may contribute to a more precise and responsive victim-centered justice framework. The aim of this study is to identify distinct victim typologies among parents who have experienced child-to-parent violence using administrative victim-support records, in order to advance a multidimensional understanding of this form of violence from a victim-centered perspective.

## Method

### Data source and procedure

The data for this study were obtained from administrative records of the victim support system from the Comunidad Valenciana (Spain), specifically from case documentation and attention summaries generated during official interventions. All datasets eligible for authorized release by the victim-support service were systematically retrieved and consolidated for analysis, in full compliance with institutional data governance frameworks and prevailing data protection regulations.

These records consist of professionals' interactions with and assessments of victims of filioparental violence from 2008 to 2024. Eligible cases comprised all records registered in the victim-support system involving parents who had experienced child-to-parent violence (filioparental violence) and were assigned a unique victim identifier. Records lacking a unique identifier, which precluded deduplication for the clustering dataset, were excluded, as were case types not corresponding to filioparental violence. For the purposes of clustering analyses, duplicate records were systematically identified and removed to ensure that each case was represented only once in the analytical dataset. Thus, te dataset included 3,834 records corresponding to 782 unique cases of parents who suffered violence, with an average of 4.9 records per case (SD = 5.3). The included variables were: *Date of Birth, Age, Sex/Gender, Marital Status, Employment Status, Economic Situation, Education Level, Number of Children, Vulnerability Factor- Offense Order/Sequence, Time of Offense, Location of Offense, Weapon Use, Physical Harm, Psychological Harm, Economic Harm, Social Harm, Perpetrator Age, Minor Perpetrator, Type of Criminal Act, and Victim-Perpetrator Relationship*.

### Participants

Victims were predominantly female (79.0%, *n* = 618), with males comprising 21.0% (*n* = 164) of the sample. The mean age of victims was 60.4 years (SD = 15.5), ranging from 32 to 87 years. In terms of marital status, 38.5% (*n* = 301) were married and 20.2% (*n* = 158) were separated or divorced, while 18.5% (*n* = 145) were widowed. Economic vulnerability was notable, with 37.6% (*n* = 294) reporting low economic status and 24.9% (*n* = 195) medium economic status, while only 5.9% (*n* = 46) reported high economic status. Educational attainment was generally low, with 31.3% (*n* = 245) having completed only primary education, 12.9% (*n* = 101) secondary education, and 4.2% (*n* = 33) higher education. On average, victims had 2.1 children (SD = 1.2).

With regards to perpetrator characteristics, when age data were available (*n* = 206), the mean age of perpetrators was 28.9 years (SD = 12.0), with ages ranging from 14 to 57 years. Minor status was specifically documented in 36 cases (4.6% of the cluster analysis sample), reflecting a subset where perpetrator age information was systematically recorded, while the majority of cases (95.4%, *n* = 746) had no documented minor status information.

### Method and data analysis

This study used a retrospective observational approach, conducting a secondary analysis of pre-existing administrative victim-support records collected during routine institutional practice. All data were anonymized to remove personal identifiers prior to analysis. In cases where records included multiple sentences per case, these were aggregated or treated as separate observations depending on analytical needs. For quantitative clustering, key variables were extracted or coded from the interaction with the responsible in the office of victim support.

Clustering was performed using K-means and the optimal number of clusters was determined via elbow and silhouette methods. As each individual could present multiple vulnerability factors, the average number of records per person was 5.69. For clustering purposes, duplicate records were removed by victim identifier, ensuring each case was represented only once in the analysis. However, the full dataset was retained for the calculation of prevalence rates of vulnerability factors.

With regards to missing data analysis and treatment, a comprehensive assessment of missing data was conducted across 22 variables. Six variables exhibited missingness, with an average of 17.0% among those affected. The highest proportion of missing values was observed in *perpetrator age*, with 71.3% missing, followed by *number of children* (11.3%), *time of offense* (11.3%), *location of offense* (3.1%), *victim age* (3.1%), and *birth date* (1.7%).

A Clustering Procedure was employed afterwards. K-means clustering was employed to identify distinct profiles of victim vulnerability. All variables were standardized using *StandardScaler* to ensure equal contribution to the clustering process. The optimal number of clusters was determined to be *K* = 2, based on both clinical interpretability and silhouette coefficient analysis. This configuration yielded the best balance between cohesion and separation while maintaining practical relevance for intervention planning.

To examine differences between clusters, we focused primarily on indicators of psychological vulnerability, as these factors are theoretically closer to the lived impact of the abusive experience and more directly informative for risk profiling and intervention than static sociodemographic characteristics. Accordingly, bivariate analyses were conducted to assess cluster differences across psychological, situational, and harm-related variables, with sociodemographic variables examined for contextual purposes only. Categorical variables were analyzed using chi-square tests, applying corrections for low expected frequencies where appropriate, while continuous variables were assessed using the Mann–Whitney U test due to non-normal data distributions. All analyses were conducted using Python 3.11, with the scikit-learn library for clustering, pandas for data handling, and matplotlib/seaborn for visualization. Statistical significance was set at α = 0.05.

## Results

**Figure 1:**
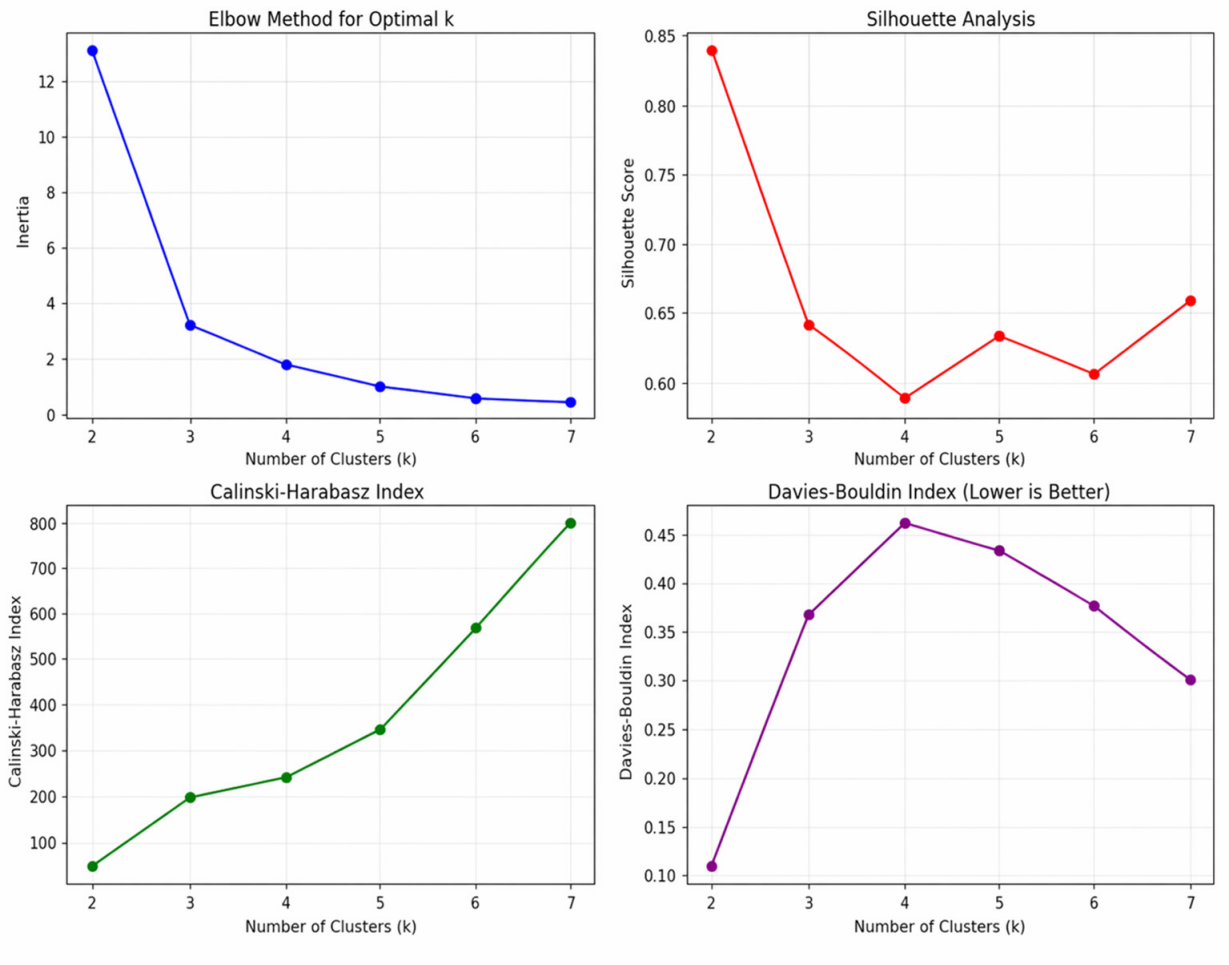
*Cluster validation metrics for optimal K selection*. Cluster validation indices used to determine the optimal number of clusters (K) for the full dataset. Top-left: Elbow method showing within-cluster sum of squares (WCSS); Top-right: Silhouette analysis indicating cluster cohesion and separation; Bottom-left: Calinski–Harabasz index, with higher values suggesting better-defined clusters; Bottom-right: Davies–Bouldin index, where lower values indicate more compact and well-separated clusters. Taken together, the metrics suggest improved performance with increasing *K*, with clearer cluster structure emerging around *K* = 2.

The cluster validation analysis revealed that *k* = 2 provides the optimal clustering solution. The silhouette score reached its maximum value of 0.840 at *k* = 2, indicating excellent cluster separation and cohesion. Similarly, the Davies-Bouldin index achieved its minimum value of 0.099 at *k* = 2, confirming minimal inter-cluster similarity. While the elbow method indicated a possible solution at *k* = 3, multiple validation indices and considerations of interpretability supported the selection of a two-cluster solution as the most robust and meaningful representation of the data. The Calinski-Harabasz index, while continuing to increase with higher *k* values, showed diminishing returns beyond *k* = 2 (Figure 1).

**Figure 2:**
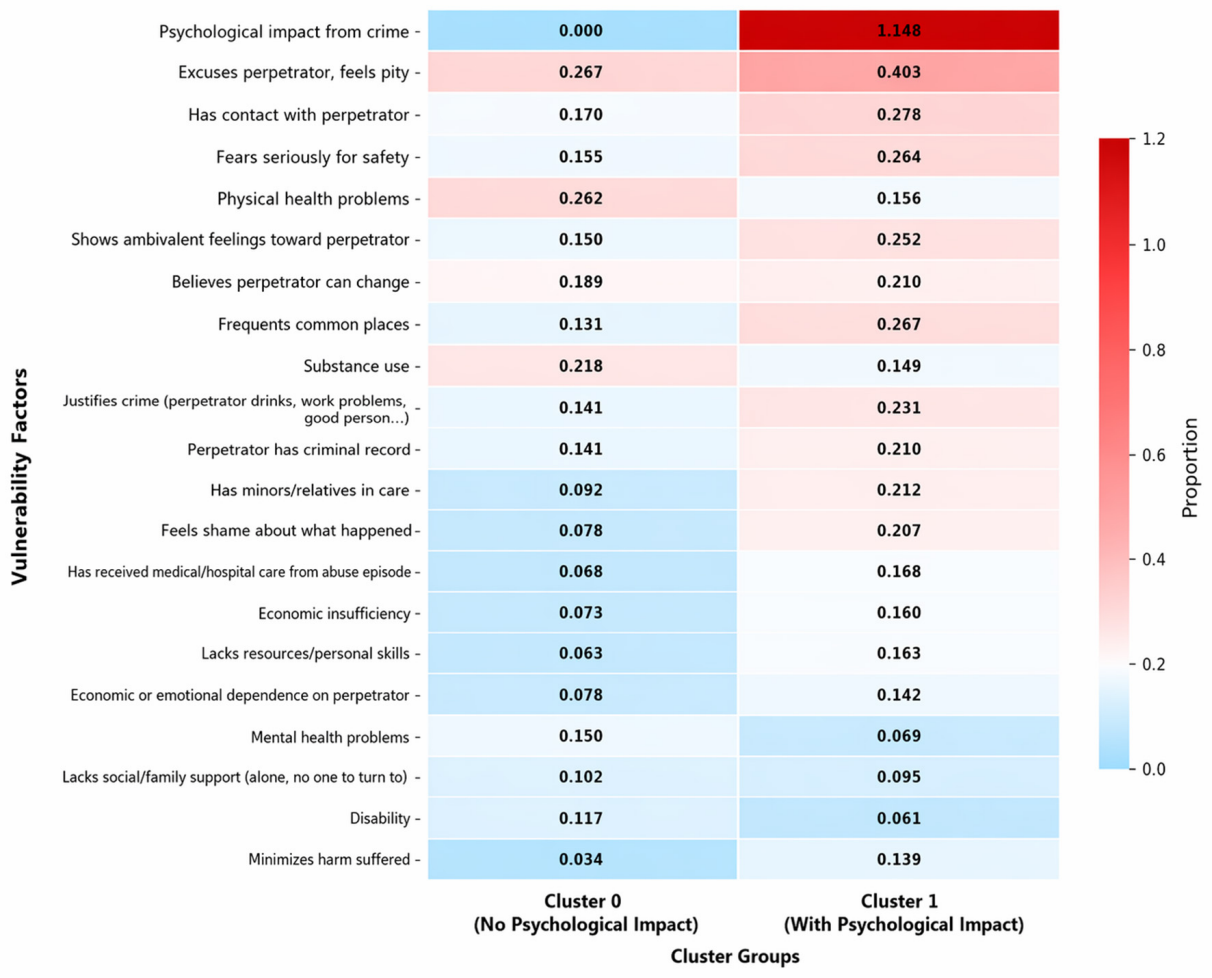
Distribution of most relevant vulnerability factors (over 0.10 proportion in at least one cluster) across identified victim clusters.

The heatmap visualization clearly demonstrates the distinctive nature of the clustering solution. Cluster 1 is defined exclusively by psychological impact, representing individuals who reported experiences of psychological violence. In contrast, Cluster 0 encompasses all other vulnerability factors with varying prevalence, and notably, does not mainly recognize cases of psychological violence. This distinction highlights the central role of psychological harm in differentiating the two groups and underscores the specificity of Cluster 1 as a psychologically impacted subgroup (Figure 2).

The vulnerability patterns observed across clusters suggest that psychological impact plays a central role in differentiating profiles of parents exposed to child-to parent violence. Rather than being defined by isolated characteristics, the high vulnerability cluster is characterized by the accumulation of psychological distress, fear, ambivalence toward the perpetrator, and ongoing relational exposure, often accompanied by health and resource-related difficulties. This clustering of vulnerabilities supports a cumulative risk perspective, whereby continued contact with the perpetrator and unresolved emotional responses may amplify the psychological burden over time. In contrast, the lower-vulnerability cluster appears to reflect situations in which the abusive experience, while present, has not translated into a comparable level of psychological or situational disruption.

With regards to sociodemographic differences, the analysis against clusters revealed statistically significant differences between variables of interest. Notably, Marital status showed a significant association with cluster membership as well (χ^2^2 = 5.42, *p* < 0.001), implying potential relational or household-level factors contributing to cluster differentiation. In this way, the key finding is that Cluster 0 has a “Widowed Profile” with 28.6% widowhood vs. Cluster1′s “Relationship Conflict Profile” with 26.9% separation/divorce rates. This suggests different vulnerability pathways: social isolation following spousal death vs. ongoing family conflict and relationship instability.

Participant age differed significantly between clusters, with the lower vulnerability group (Cluster 0) having a higher mean age (61.34 years) compared to the higher vulnerability group (Cluster 1), which had a mean age of 58.01 years (*U* = 56,993, *p* = 0.010). This age difference aligns with the higher proportion of widowed individuals observed in Cluster 0, suggesting a demographic widowed profile consistent with older victim populations. Similarly, perpetrators in Cluster 0 were older on average (mean = 31.4 years) than those in Cluster 1 (mean = 28.4 years; *U* = 5,215, *p* = 0.042). Despite statistical significance, the effect sizes were small (victim age: *r* = 0.095; perpetrator age: *r* = 0.141), indicating limited practical impact.

Occupational status also varied significantly (χ^2^2 = 2.25, *p* = 0.035), indicating potential socioeconomic disparities across clusters. In this way, Cluster 0 (“Retirement Profile”) relies mainly on pensions (51.5%) and shows lower employment (13.6% high SES jobs), and Cluster 1 (“Active Workforce Profile”) shows higher employment across all types: permanent (19.3%), temporary (5.4%), and self-employed (4.3%).

Other variables, such as sex, educational attainment, economic harm, number of children, and situational factors such as the use of weapons, location of the incident, or whether the perpetrator was a minor, did not show statistically significant differences between clusters. These findings suggest that the clustering algorithm captured robust distinctions in vulnerability profiles, particularly in psychological and physical harm domains, but that some demographic and contextual characteristics were more evenly distributed across groups.

## Discussion

The present clustering analysis provides empirical support for differentiating victim typologies among parents who have experienced filio-parental violence in adulthood. By moving beyond approaches that conceptualize victims as a homogeneous group, this study offers a more granular and context-sensitive understanding of victimization patterns (Carlson and Jones, 2010). The two-cluster solution identified here underscores the heterogeneity of victim experiences and supports calls for differentiated intervention frameworks within family violence research (Dixon and Browne, 2003; Mennicke, 2018).

Validation metrics indicated strong internal structure (silhouette score = 0.840; Davies–Bouldin index = 0.099), supporting the robustness of the typology. Cluster 1, comprising a substantial proportion of the sample, was defined primarily by pervasive psychological harm, suggesting a profile characterized by sustained relational conflict and emotional trauma. Although most individuals in this cluster were not separated or divorced, the higher prevalence of separation/divorce compared to Cluster 0 suggests relational instability as a contextual contributor rather than a defining feature.

In contrast, Cluster 0 represented an older and more demographically diverse group, characterized by the absence of psychological harm as a unifying factor. This cluster showed a higher prevalence of widowhood and a broader range of vulnerability indicators, suggesting that victimization may be shaped by life-course and contextual vulnerabilities rather than acute emotional trauma alone. These findings reinforce the systemic and longitudinal dimensions of domestic violence, particularly in older populations.

The identification of these distinct clusters has practical implications for intervention. Cluster 1, marked by widespread psychological impact, may benefit from trauma-informed therapeutic approaches focused on emotional regulation, trauma processing, and relational repair. Given the higher prevalence of separation and divorce within this group, interventions addressing relational conflict dynamics may also be warranted. Understanding emotional dysregulation and post-traumatic stress symptoms is particularly important for victims exposed to repeated episodes of violence (Muñoz-Rivas et al., 2021).

Cluster 0, by contrast, calls for a broader and more multidimensional response. The absence of self-reported psychological harm in this group must be interpreted cautiously, as it may reflect normalization of violence or reduced recognition of emotional harm. This suggests the need for further assessment and awareness strategies to prevent under-identification of psychological impact.

These findings align with broader literature emphasizing the limitations of binary victim–perpetrator frameworks in domestic violence research (Hines and Douglas, 2011). Similarly, relying solely on sex as a primary explanatory variable may obscure more complex relational and contextual dynamics (Capaldi and Langhinrichsen-Rohling, 2012). Although sex was not included in the present analyses, potential cohort effects cannot be ruled out. A more nuanced perspective that considers relationship-specific dynamics, controlling behaviors, and motivational patterns is necessary (Hine et al., 2020). Furthermore, the underrepresentation of certain victim groups, including men, in existing research may hinder the development of inclusive support services (Laskey et al., 2019). As Moore (2021) notes, lived experiences often exceed rigid categorical classifications, reinforcing the value of flexible analytical approaches.

The systemic nature of violence must also be considered. Bias-motivated aggression, rooted in identity and prejudice, illustrates how individual victimization is embedded within broader psychosocial and ecological contexts (Díaz-Faes and Pereda, 2020). While the present study does not directly examine bias-motivated violence, its findings support the broader argument that victim experiences cannot be reduced to isolated individual characteristics.

Several limitations warrant consideration. First, longitudinal designs are needed to examine how victim typologies evolve over time and to assess the long-term effects of tailored interventions. Second, future research should explore intersectional dimensions, including socioeconomic status and cultural background. A substantial proportion of perpetrator age data was missing (71.3%), limiting interpretability of age-related findings. This missingness reflects limitations of the administrative records rather than analytical procedures. Accordingly, age-related interpretations are exploratory. Additionally, perpetrator sex was not recorded in the dataset, precluding analysis of sex-related patterns and limiting demographic profiling of perpetrator characteristics. Nevertheless, the results suggest that violence against parents cannot be fully explained by isolated individual factors such as age or education. A more complex and interdisciplinary framework incorporating life trajectories, relational dynamics, and structural vulnerabilities is needed to inform differentiated intervention strategies.

## Conclusions

This study provides empirical support for the existence of differentiated victim typologies among parents who have experienced filio-parental violence in adulthood. The two-cluster solution identified distinct patterns of psychological harm and contextual vulnerability, highlighting that victimization in this context cannot be treated as a homogeneous phenomenon.

The findings underscore the importance of adopting a victim-centered and context-sensitive approach to intervention. While one group was primarily defined by pervasive psychological impact and relational instability, the other reflected more heterogeneous vulnerability patterns shaped by life-course and contextual factors. These distinctions suggest that support mechanisms and justice responses should be tailored to the specific profiles and needs of affected parents rather than relying on uniform intervention models. Moreover, the results reinforce the need for multidimensional frameworks capable of capturing the complexity of filio-parental violence in adulthood. Integrating data-driven typologies into institutional practice may enhance the precision of assessment, improve allocation of support resources, and contribute to more responsive and individualized intervention strategies.

## Data Availability

The data analyzed in this study is subject to the following licenses/restrictions: vulnerability of the victims. Requests to access these datasets should be directed to mariacarmen.moret@ucv.es.
